# Rhodamine-Functionalized Mechanochromic and Mechanofluorescent Hydrogels with Enhanced Mechanoresponsive Sensitivity

**DOI:** 10.3390/polym10090994

**Published:** 2018-09-06

**Authors:** Lijun Wang, Wanfu Zhou, Quan Tang, Haiyang Yang, Qiang Zhou, Xingyuan Zhang

**Affiliations:** 1 CAS Key Laboratory of Soft Matter Chemistry, School of Chemistry and Materials Science, University of Science and Technology of China, Hefei 230026, China; ljwang2@mail.ustc.edu.cn (L.W.); tangq@mail.ustc.edu.cn (Q.T.); zq199532@mail.ustc.edu.cn (Q.Z.); 2 Oil Production Technology Institute, Daqing Oilfield Company Ltd., Daqing 163453, China; zhouwf@petrochina.com.cn

**Keywords:** rhodamine mechanophore, micellar hydrogel, mechanochromic, mechanofluorescent, mechanoresponsive sensitivity, stress/strain detection

## Abstract

Smart materials responsible to external stimuli such as temperature, pH, solvents, light, redox agents, and mechanical or electric/magnetic field, have drawn considerable attention recently. Herein, we described a novel rhodamine (Rh) mechanophore-based mechanoresponsive micellar hydrogel with excellent mechanochromic and mechanofluorescent properties. We found with astonishment that, due to the favorable activation of rhodamine spirolactam in the presence of water, together with the stress concentration effect, the mechanoresponsive sensitivity of this hydrogel was enhanced significantly. As a result, the stress needed to trigger the mechanochromic property of Rh in the hydrogel was much lower than in its native polymer matrix reported before. The hydrogel based on Rh, therefore, exhibited excellent mechanochromic property even at lower stress. Moreover, due to the reversibility of color on/off, the hydrogel based on Rh could be used as a reusable and erasable material for color printing/writing. Of peculiar importance is that the hydrogel could emit highly bright fluorescence under sufficient stress or strain. This suggested that the stress/strain of hydrogel could be detected quantificationally and effectively by the fluorescence data. We also found that the hydrogel could respond to acid/alkali and exhibited outstanding properties of acidichromism and acidifluorochromism. Up to now, hydrogels with such excellent mechanochromic and mechanofluorescent properties have rarely been reported. Our efforts may be essentially beneficial to the design of the mechanochromic and mechanofluorescent hydrogels with enhanced mechanoresponsive sensitivity, fostering their potential applications in a number of fields such as damage or stress/strain detection.

## 1. Introduction

In recent decades, smart materials in response to external stimuli such as temperature, pH, solvents, light, electric/magnetic field, and redox agents, have attracted widespread attention [1,2]. Among the various fascinating responsiveness, materials sensitive to force are quite interesting and important due to their ability to report damage [3,4,5], sense stress/strain [6,7,8,9,10], strengthen mechanical properties [11,12,13,14], and release small molecules [15,16]. Outstandingly, mechanochromic polymers (MCP), which can change color when subjected to external force, as one important kind of mechanoresponsive materials, have been widely used to monitor stress or strain [17]. Currently, most MCP materials are prepared by covalently incorporated specific mechanophores into polymer backbone. The relative mechanophores have been well-studied, including spiropyran (SP) [3,6,7], rhodamine (Rh) [18,19,20], diarybibenzofuranone unite [21,22,23,24], dioxetane unite [8,25,26,27], and hexaarylbiimidazole unite [28], and they have been successfully incorporated into a range of materials such as elastomers [18,25,29,30,31], latex coating [32], micelles [33], and microgels [34]. However, due to the poor solubility of the mechanophores in aqueous solutions, and especially the limited stress/strain the mechanophores loaded, mechanochromic hydrogels based on specific mechanophores have been rarely reported. Only very recently, by covalently incorporating SP into the networks based on the copolymer of methyl acrylate and acrylamide, was a novel mechanochromic and mechanically strong micellar hydrogel successfully developed. The key to success relied on the fact that the stress the hydrogels loaded was intensively concentrated on SP, the crosslinker of the networks, triggering the mechanochromic property of the hydrogels [35]. However, the photostability of SP is limited, mainly due to the photodegradation of its isomer merocyanine (MC, a ring-opened and colored form), and, with photobleaching properties, the SP mechanophore applied in the fields of sensing stress or strain has poor reversibility and self-regenerative ability [36,37]. Moreover, the MC form of the SP mechanophore shows an extremely low fluorescence quantum yield (Фf < 0.02) [38,39]; as a result, the quantitative detection of damage or stress/strain by fluorescence measurement is difficult [7,19,30,40]. These disadvantages abovementioned have serious negative impacts on the applications of SP-based hydrogels as mechanical sensors.

**Scheme 1:**
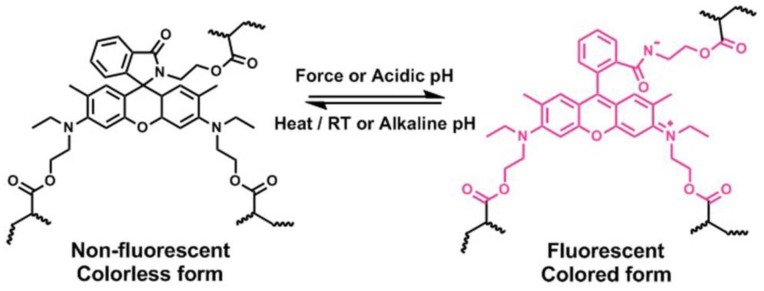
Force- or acid-induced ring-opening of rhodamine (Rh)-based mechanophore covalently incorporated into polymers. The structural transformation between spirocyclic form and ring-opening form is reversible.

Rh derivatives, one of the effective mechanophores reported in the recent literature [18,19,20], could proceed force-induced color change when their structure changed from ring-closed structure to a ring-opened zwitterion, accompanying with the red shifts of the absorption band and emission of fluorescence (Scheme 1). It is already known that Rh is an outstanding fluorescent dye, exhibiting excellent optical properties of stability, high absorption coefficient, and long wavelength absorption and emission [41,42,43]. More importantly, its quantum efficiency is very high (Фf ≈ 0.67), which is about 2 orders of magnitude higher than that of MC [19,41,43]. If these kinds of mechanophores were incorporated into hydrogels, the hydrogels would also exhibit mechanochromic properties; moreover, they could emit bright fluorescence, distinctly different from the SP-based hydrogels. Since fluorescence signal output is ratiometric, and fluorescent detection, as a self-referencing two-band comparative detection method, is basically free from external interference, Rh-based hydrogels with excellent mechanofluorescent properties could be used to quantificationally and effectively detect damage or stress/strain. The Rh mechanophore first reported by Jia group was covalently linked with a crosslinked polyurethane network [18] and there existed a big disadvantage for the activation of this mechanophore, in that huge pressure was needed to activate the mechanophores, that is to say, its mechanical sensitivity was quite low. Another Rh-based mechanophore was later developed by the Bai group [19]; when this was embedded in the single- or double-network elastomer, upon stretching, the mechanical response degree of Rh was very low. When it was incorporated into a triple-network elastomer, the mechanical sensitivity of the elastomer was enhanced. However, for this system, to achieve a high degree of mechanical response, high stress was still needed. In a word, the activation of Rh mechanophores is very difficult, except when sufficient mechanical force has been applied.

**Figure 1:**
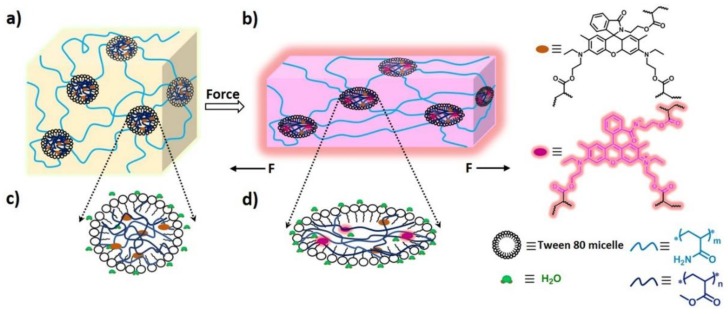
Illustration of the mechanical activation of Rh mechanophore incorporated into the micellar hydrogels. (**a**) Micellar hydrogels with serious chain entanglement before stretching; (**b**) mechanochromic hydrogels (purple/red color and pink/rose red fluorescence) with chain orientation; (**c**) microspheres with serious chain entanglement containing small amount of water; (**d**) deformed microspheres with Rh mechanophore activation facilitated by chain orientation and water.

Since micellar hydrogels as reported previously [35] exhibited excellent mechanical strength and high fracture strain, adopting this kind of hydrogel structure, the mechanoactivation of Rh may be enhanced a lot, and Rh-based hydrogels may exhibit outstanding mechanochromic and mechanofluorescent properties (Figure 1). This is because high strain could make the polymer chains reach a high alignment state resulting in the enhancement of mechanophore activation [6,19,29,44,45] and high strength could greatly promote the reaction of Rh from ring-closed form to ring-opened form [3,32,35,46]. More importantly, the special microspheres within the hydrophilic hydrogels were not completely free of water [47,48,49], and water was beneficial to the mechanical activation of Rh mechanophores [20]. In addition, since Rh spirolactam derivatives exist commonly in a colorless and nonfluorescent form in neutral or alkaline solvents, and they can structurally equilibrate to a colored and fluorescent ring-opened form upon an acidic pH [50,51,52], Rh-based hydrogels may also respond to acid and alkali.

Herein, we adopted a micellar-copolymerization method to fabricate a novel Rh-based mechanoresponsive hydrogel. Rh as a crosslinker was firstly dissolved in methyl acrylate (MA) and then in a TWEEN 80 aqueous solution to form a uniform emulsion; after the addition of acrylamide (AAm), followed by photoinitiated polymerization, the Rh-crosslinked hydrogels were obtained. The hydrogels exhibited outstanding mechanochromic and mechanofluorescent properties, and within the hydrogels, the mechanoresponsive sensitivity was enhanced a lot; as a result, the stress needed to trigger the mechanochromic property of Rh in hydrogels was much lower than in its native polymer matrix reported before. Of peculiar importance, with highly bright fluorescence induced by force, the stress/strain could be detected quantificationally and effectively. Moreover, the Rh-based hydrogels also exhibited outstanding properties of acidichromism and acidifluorochromism. This double stimuli-responsive system will enhance the multipurpose applications of soft materials, and they will be of particular interest for biological sensors and imaging.

## 2. Materials and Methods

### 2.1. Materials

Unless otherwise stated, all starting materials were obtained from commercial suppliers and used without purification. Acrylamide (AAm, 99%, Merck, Shanghai, China), methyl acrylate (MA, 99%, Merck), dodecyl 2-methylacrylate (C12, 97%, Aladdin, Shanghai, China), stearyl methacrylate (C18, 96%, Aladdin), ethylene glycol dimethacrylate (EGDMA, 98%, Aladdin), 1-hydroxycyclohexyl phenyl ketone (HCPK, Photo initiator 184, >99%, Aladdin), TWEEN 80 (CP, Merck), and pyrene (Py, 97%, Aladdin) were used as received. Water used in this work was purified by a Millipore water-purification system with a resistivity of 18.2 M cm (H_2_O, pH = 6.8), and water solutions with different pH values (1.0, 3.0, 5.0, 5.5, 13.0) were prepared.

### 2.2. Synthesis and Preparation

#### 2.2.1. Synthesis of Rh Crosslinker

The Rh crosslinker were synthesized via a multistep organic synthesis as reported previously (Scheme S1) [19]. The chemical structure and purity of Rh crosslinker were identified by ^1^H NMR (as shown in Figure S1).

#### 2.2.2. Preparation of Rh-Based Hydrogels

Several samples of mechanophore crosslinked hydrogels with various solid contents and crosslinker concentrations were prepared, and they were listed in Table S1.

Taking MA_35_-Rh_0.5_-AAm_35_ as an example, a typical hydrogel-preparation procedure is as follows. Rhodamine mechanophore crosslinker (33.6 mg, 0.5 mol% of MA) and the HCPK (Photo initiator 184, 6.8 mg, 0.2 mol% of MA) were dissolved in methyl acrylate (0.7 g, =35 wt%). The solution was then dissolved in TWEEN 80 aqueous solution (50 µL/0.6 mL, =30 wt%) and vortexed for 5 min to form a uniform emulsion. Then, acrylamide (0.7 g, 35 wt%) was added to the emulsion, followed by vortex mixing for another 5 min. After bubbling N_2_, the mixture was then injected into a glass mold with a 1.5 mm thick Teflon spacer and exposed to UV light (4 mW/cm^2^). The gel was formed after 4 h photopolymerization.

#### 2.2.3. Preparation of the Control Hydrogels Containing Py

Five samples of crosslinked hydrogels similar to those above were prepared, which adopted EGDMA instead of Rh as crosslinker, with the addition of pyrene, they were used to describe the polarity of the micellar structures within the hydrogels. The concentration of pyrene was 1 × 10^−6^ M for each sample. The detailed hydrogel preparation procedure was the same as that of Rh-based hydrogels, and their detailed compositions were listed in Table S3.

#### 2.2.4. Preparation of Poly(Methyl Acrylate) Containing Py

Pyrene and the HCPK (Photo initiator 184) were dissolved in methyl acrylate. After bubbling N_2_, the mixture was exposed to UV light (4 mW/cm^2^). The PMA was obtained after 4 h photopolymerization.

### 2.3. Characterizations

#### 2.3.1. Mechanical Characterization

Tensile and loading–unloading tests of the tough hydrogel were measured by an electrical universal material-testing machine with a 500 N load cell (EZTest, SHIMADZU, Kyoto, Japan). The samples were cut into a long dumb-bell shape with a gauge length of 13 mm, a width of 2.2 mm, and a thickness of 1 mm. The crosshead velocity was kept at 60 mm min^−1^ during the normal tensile tests. As for the successive loading–unloading tests, the crosshead velocity was kept at 90 mm min^−1^ for both. In addition, silicone oil was coated on the hydrogel surface during the successive loading–unloading tests. When the tests finished, the tested hydrogel was coated with a layer of silicone oil, encased by the plastic wrap and stored in a sealed plastic pipe to avoid water volatilizing. All the measurements were carried out at room temperature (about 20 °C).

#### 2.3.2. Digital Image Analysis

Optical images were taken by a NIKON D7000 camera (NIKON, Tokyo, Japan), and the mechanical activation of the Rh crosslinker in the hydrogels was analyzed using RGB color analysis. A whiteboard was used as the background under ambient room light conditions. The photos were analyzed in Adobe Photoshop CS6 Software (Adobe, San Jose, CA, USA). After the background was white-balanced, the average intensity of the red (R), green (G), and blue (B) channel at the region of interest was obtained. The corrected RGB value of the gel specimen center was converted to the (x, y) value for the chromaticity diagram via the website (http://www.colorhexa.com). The color of each gel specimen was subsequently located in the chromaticity diagram (CIE 1931).

#### 2.3.3. Fluorescence Spectrometer

Fluorescence spectra measurements were performed on a Shimadzu RF-5301PC spectrofluorophotometer (Shimadzu Corporation, Kyoto, Japan), and the excitation wavelength was 365 nm.

#### 2.3.4. Differential Scanning Calorimetry (DSC) Measurements

DSC measurements were carried out on TA Instruments DSC Q2000 (TA Instruments, New Castle, DE, USA) under a nitrogen atmosphere. The gel samples were cut into small specimens of about 10 mg weight and sealed in aluminum pans. Then, they were scanned between 0 and 60 °C with a heating and cooling rate of 5 °C·min^−1^.

## 3. Results and Discussion

### 3.1. Mechanical Properties of Rh-Functionalized Hydrogels

To fabricate model hydrogels embedded with hydrophobic Rh mechanophores in hydrated polymer network, a micellar-copolymerization method as previously reported [35] was adopted. A series of poly(AAm-co-MA/Rh) hydrogels with different solid contents from 50 to 70 wt%, and different mechanophore crosslinker concentrations from 0.3% to 0.6% were prepared, as shown in Table S1, from which we want to obtain the hydrogels with good responsiveness to mechanical stress.

**Figure 2:**
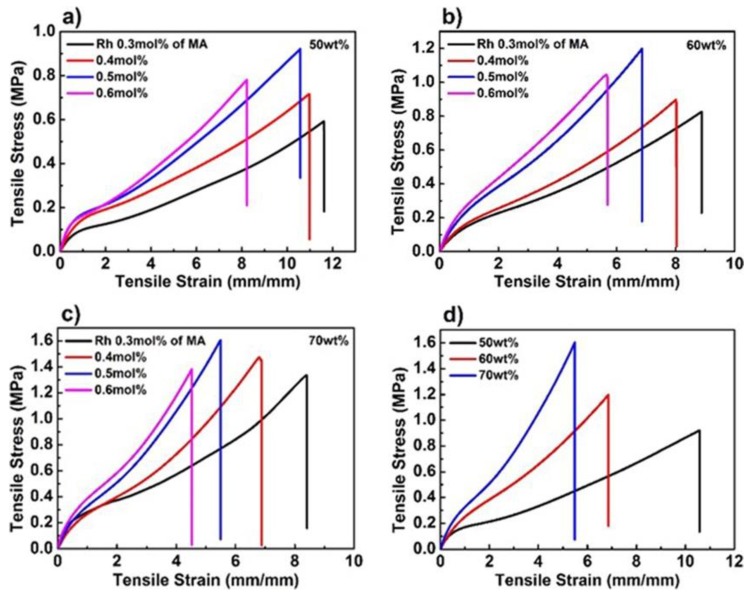
Mechanical properties of the poly(AAm-co-MA/Rh) hydrogel. (**a**) Tensile stress–strain curves of poly(AAm-co-MA/Rh) hydrogels with a solid content of 50 wt%, (**b**) 60 wt%, (**c**) 70 wt%, and Rh-crosslinker concentrations of 0.3, 0.4, 0.5, and 0.6 mol% of methyl acrylate (MA), respectively. (**d**) Tensile stress–strain curves of poly(AAm-co-MA/Rh) hydrogels with a solid content of 50, 60, 70 wt% and the same Rh crosslinker concentration of 0.5 mol% of MA.

Since the concentrations of mechanophore crosslinkers had great effect on the mechanical properties of hydrogels, and further greatly affected the mechanical response degree of Rh [3,32,35]. For the Rh-based hydrogels with different mechanophore-crosslinker concentrations, we examined their mechanical properties through a series of tensile tests. In Figure 2 and Table S2, with the increase of Rh concentrations regardless of solid contents, the tensile strength (*σ*_f_) firstly increased from 0.3% to 0.5% and then decreased from 0.5% to 0.6%, while the fracture strain (*ε*_f_) monotonically decreased. In parallel, the elastic modulus (*E*) approximately unidirectionally increased. As for the highest concentration of 0.6 mol%, its tensile strength (*σ*_f_) and fracture strain (*ε*_f_) both decreased dramatically. It can be learned that the concentrations of the Rh crosslinker indeed greatly affected the mechanical property of the hydrogels. Similar to the conventional crosslinking system, within an appropriate range of crosslinking concentration, the mechanical strength and stiffness of the Rh-crosslinked micellar hydrogel was improved with the increase of the crosslinker concentration. While the concentration was further increased, the hydrogel exhibited enhanced brittleness, leading to inferior mechanical strength and poor toughness. Thus the hydrogel here with an Rh-crosslinker concentration of 0.5 mol% exhibited the highest mechanical strength and large-enough fracture strain (*ε*_f_), which was beneficial to the mechanical activation of Rh. Moreover, hydrogels with higher mechanophore concentration appeared to be more sensitive to stress, and when the tensile stress/strain was enough, more mechanophores were activated [32,44,45]. Thus, when the solid content was fixed, hydrogels with an Rh-crosslinker concentration of 0.5 mol% were the better option to study the mechanical activation of Rh.

According to previous work [35], solid contents also had important influence on the mechanical properties of hydrogels. According to Figure 2 and Table S2, with the increase of solid content from 50% to 70%, the tensile strength (*σ*_f_) increased from 0.92 to 1.60 MPa, and the elastic modulus (*E*) increased from 419 to 632 kPa, while the fracture strain (*ε*_f_) decreased from 10.5 to 5.6. With relatively high mechanical strength and large enough fracture strain (*ε*_f_), these hydrogels were used to study the mechanoactivation behavior of the Rh mechanophores.

### 3.2. Mechanochromic Behavior of Rh-Functionalized Hydrogels

**Figure 3:**
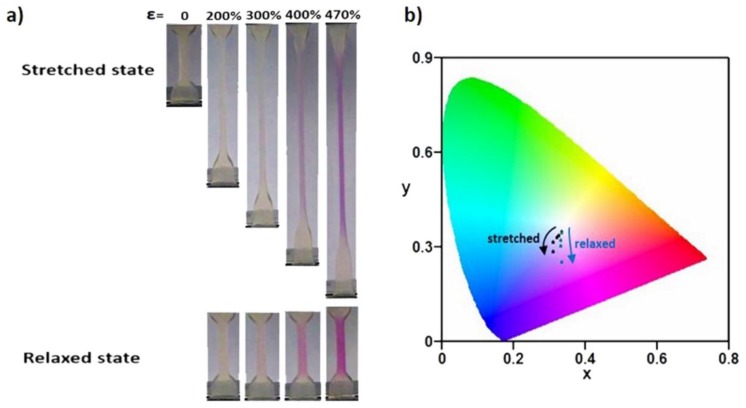
(**a**) Optical images of poly(AAm-co-MA/Rh) with a solid content of 70 wt% at both stretched state and the corresponding relaxed state. (**b**) The x, y chromaticity diagram for the color-change pathways of both stretched gels at loading state and relaxed gels at unloading state (standard CIE 1931). Note: *ε* means stretch ratio.

Surprisingly, all of the hydrogels exhibited mechanochromic properties. In Figure S2 and Figure 3a and Video S1, the optical images of these hydrogels at both the loading state and unloading state are displayed, and their corresponding stress–strain curves were obtained as shown in Figure S3. For the hydrogels with a solid content of 50 wt%, the color change was difficult; only when the stretching ratio reached 500%, did the gel begin to show color change between loading and unloading states (Figure S2a), and the corresponding stress was as low as 0.385 MPa (Figure S3a). This meant that the stretching force was relatively low but large enough to transfer to the Rh mechanophores, and the ring-opening reaction of the spirolactam of Rh was triggered. When the gels were stretched to three to nine times their original length, obvious dynamic color changes with increasing pink were observed. As for the 60 wt% hydrogel, the slightly pink coloration started to emerge after 300% strain (Figure S2b), and the corresponding stress was 0.536 MPa (Figure S3b). Moreover, the red coloration deepened with the increasing strain; interestingly, at 500% strain, the color of the gel was dramatically redder. Further increasing the solid content to 70 wt%, the coloration of the gel when it was stretched to 470% turned to purple (Figure 3a), which indicated more mechanophore molecules were activated, and the corresponding stress was as high as 1.33 MPa (Figure S3c). By comparing the imaging pictures, it can be qualitatively demonstrated that the mechanical response degree of Rh increased with the increase of strain, and, with the increase of solid content, the mechanical strength was enhanced; at the same time, the mechanical response degree of Rh at a certain high strain increased as well. Moreover, the color changes in the gauge section of digital images of gels were analyzed by using the RGB color channels (Figure 3b). Taking 70 wt% gel as a typical representation, the x, y chromaticity diagram for the color-change pathways of both stretched gels and relaxed gels was calculated. Upon loading, as the stretch ratio increased, the chromaticity coordinates of the gel showed a cambered pathway from the pale yellow region to the light purple region. When the stress was removed, the relaxed gel showed a linear pathway toward the purple-red color.

### 3.3. Mechanofluorescent Property of Rh Functionalized Hydrogels and Fluorescent Detection of Stress/Strain

**Figure 4:**
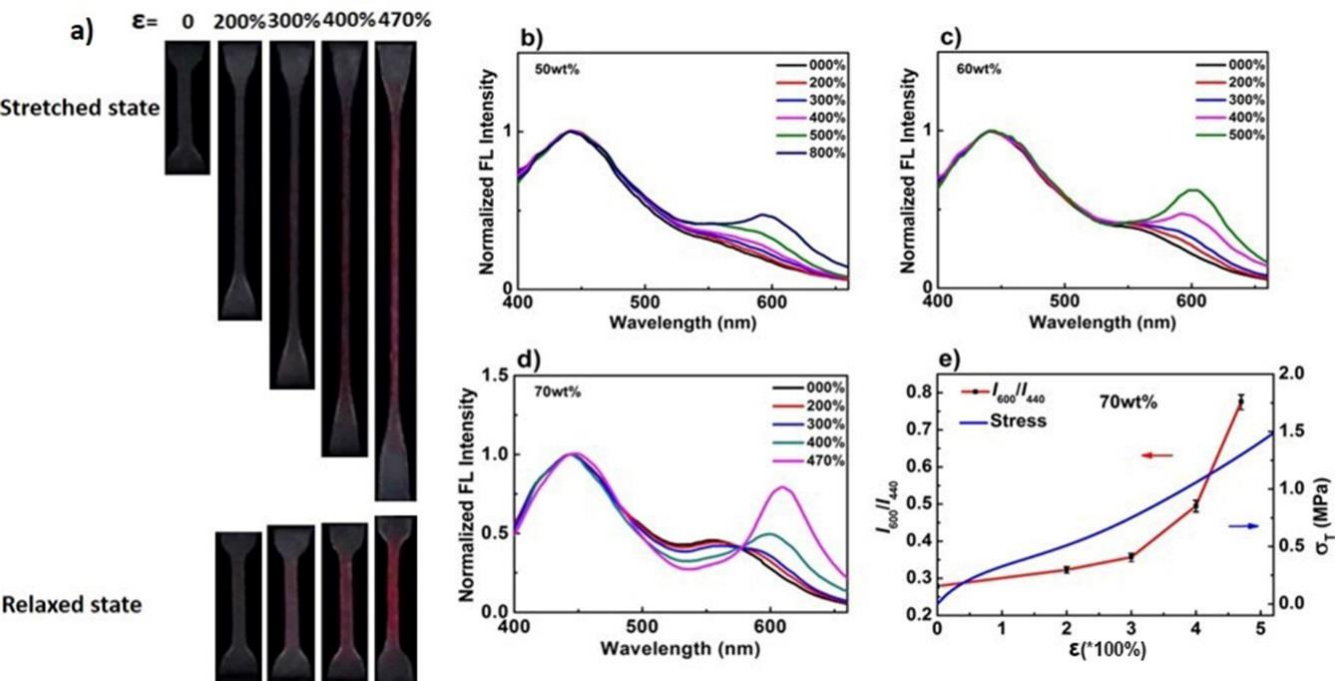
(**a**) Fluorescent images of poly(AAm-co-MA/Rh) hydrogel with a solid content of 70 wt% at both stretched state and the corresponding relaxed state. Emission spectra of the hydrogel with a solid content of (**b**) 50 wt%, (**c**) 60 wt%, and (**d**) 70 wt% recorded at increasing strain levels during uniaxial stretching. (**e**) Stress and fluorescence-intensity ratio *I*_600_/*I*_440_ as a function of stretch ratio for the hydrogel with a solid content of 70 wt%.

The hydrogels also exhibited mechanofluorescent properties; under mechanical stress, the hydrogels presented a fluorescent color change from pale yellow to rose red, as shown in Figure 4a, fluorescent color images of poly(AAm-co-MA/Rh) hydrogel with a solid content of 70 wt% was taken at both stretched state and relaxed state. In agreement with visible color changes, the fluorescent color emerged to be red at 300% strain, and the red color deepened with further elongation up to 470% strain, while the gels after relaxing from stretching stress held a pink color instead of yellow, which was different from that described in previous work [19], it was assumed to be related to the residual network deformation and potential stress. Notably, with bright fluorescence triggered by force, the hydrogels could be used as a fluorescent probe to quantificationally and effectively detect stress/strain or damage.

The fluorescence-emission spectra were later recorded during tensile loading (Figure 4), and we used them to further accurately analyze the mechanical response degree of Rh in the hydrogels. On the fluorescence-emission spectra, two peaks were identified at 420 nm and 520–625 nm, which we associated with the emission of the closed form and the isomerous form of Rh, respectively. With the continuous increase of the stretch ratio, a new band, approximately centered at 600 nm, slowly enhanced, indicating that the planar zwitterions induced by force were formed. Thus, the variation of fluorescence-intensity ratio *I*_600_/*I*_440_ could be used to quantitatively characterize the mechanical response degree of Rh [19]. As for the 50 wt% hydrogel, the fluorescence intensity at 600 nm increased obviously slower than the other two (Figure 4b). When the stretch ratio reached 800%, the corresponding stress was only 0.67 MPa, and the fluorescence-intensity ratio was just about 0.466 (Figure S4a). When the solid content increased to 60 wt%, the fluorescence intensity at 600 nm increased obviously with the increase of stretch ratios (Figure 4c), and the fluorescence intensity ratio reached about 0.62 when the strain was 500%, while the tensile stress was 0.83 MPa (Figure S4b). Fluorescence intensity increased more obviously for the 70 wt% gel, and, when the strain reached 470%, the emission peak generated a bathochromic shift (Figure 4d), and the corresponding fluorescence-intensity ratio increased dramatically to 0.776, though the stress needed increased to 1.30 MPa (Figure 4e). Based on the relationships of stress, fluorescence-intensity ratio, and stretch ratio, which were clearly shown in Figure 4e and Figure S4, the correspondence between the mechanical response degree of Rh and stress/strain could be acquainted quantificationally. In addition, as shown in Figure 3 and Figure 4a, and Figure S2, when the stretching ratio was 200%, the gels did not show an obvious and visible color change or a fluorescent color change between the loading and unloading states. Nevertheless, from Figure 4b–d, we can see that the emission intensity at 600 nm increased in fact, even though it was small, which meant a small amount of Rh was actually activated by force. It clearly verified that the detection of stress or strain through the quantificational calculation of fluorescence data was more accurate and effective than that through the analysis of qualitative color changes. Based on this point, the Rh-based hydrogels here had a great advantage over the SP hydrogels.

### 3.4. Erasable Writing Properties of Rh-Functionalized Hydrogels

**Figure 5:**
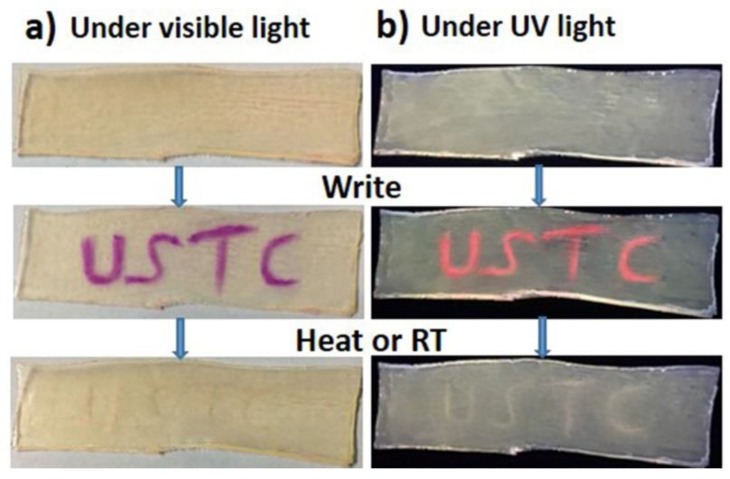
The abbreviation of our university, “USTC”, was written on the hydrogel, and it was erased by heating or at room temperature automatically. (**a**) Images taken under visible light; (**b**) fluorescent images taken under UV light.

Considering the hydrogels exhibited good mechanochromic and mechanofluorochromic properties, they could be used as mechanochemical writing/drawing tablets. As shown in Figure 5, the word “USTC” could be easily written on the gel by a pencil-like club (Figure S5), and it exhibited a deep purple color by the naked eye; in parallel, it could also exhibit rose-red fluorescent color under 365 nm UV light. Such writing with deep-purple and red-fluorescent color can be easily erased by heating it at 50 °C for 15 min or putting it at room temperature over 24 h automatically, which was verified by the dynamic fluorescent spectra of the hydrogels after stress was released (Figure S6 and Part S1). In addition, the visible/fluorescence color change between red and colorless could be repeatedly switched back and forth by cycling stress loading and stress unloading, which could be spectroscopically quantified (Figure S7 and Part S1). Therefore, the hydrogels described here could serve as reusable writing and erasing materials.

### 3.5. Enhanced Mechanoresponsive Sensitivity within the Micellar Hydrogels

Since the fluorescence-intensity ratio *I*_600_/*I*_440_ increased with the continuous activation of the Rh mechanophore, we here used the increment of *I*_600_/*I*_440_ to represent the mechanical response degree of Rh. Combined with Figure 4d,e, taking 70 wt% gels as example, through the analysis of the emission spectra and the relationships of stress, fluorescence intensity, and stretch ratio, we learned that when the stretch ratios reached 200% or 300%, the fluorescence-intensity ratios increased tinily, from 0.28 to 0.32 or 0.36, the increment was 0.04 or 0.08, and the corresponding stress was 0.50 and 0.75 MPa, respectively. Compared with the organic elastomers reported in previous work [19], even for the triple-networks elastomer with relatively higher mechanical sensitivity, of which the mechanophore concentration was higher than those of the hydrogels fabricated in this work, when it was applied with stress almost over 2 MPa, the increment of fluorescence-intensity ratio was very small and less than 0.1. Furthermore, with the further increase of stretch ratios to 400% and 470%, the fluorescence-intensity ratios increased dramatically, from the original 0.28 to 0.50 and 0.78, the increment was 0.22 and 0.5, and the corresponding stress was 1.05 and 1.30 MPa, respectively. In comparison, the fluorescence-intensity ratios of Rh mechanophores in the elastomer could increase from 0.1 to 0.3 and 0.6 (the increment of fluorescence-intensity ratios was almost the same as those of the 70 wt% gels with stretch ratios of 400% and 470%), and their corresponding stress reached about 2.5 MPa and 5.8 MPa, respectively, which were extremely higher than those of the hydrogels. It can be learned that, to reach the same mechanical response degree of Rh, the stress needed for the hydrogels was quite lower than that of the elastomers, so the mechanoresponsive sensitivity of hydrogels here was much higher than that of the elastomers.

**Figure 6:**
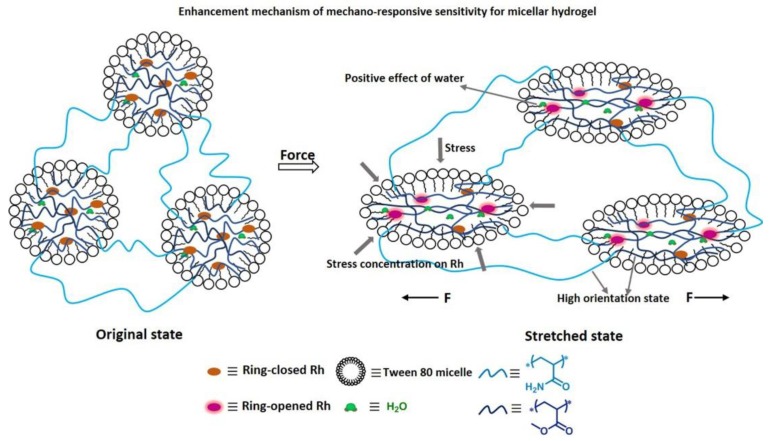
The enhancement mechanism of mechanoresponsive sensitivity for micellar hydrogel: (1) water favoring the activation of Rh; (2) stress concentration on Rh promoting the cleavage of the linkages in the Rh; (3) easy to reach high orientation state for the Rh-crosslinked chains contributing to the mechanoactivation of Rh.

The probable enhancement mechanism for the hydrogel compared with the elastomer was as follows (Figure 6). Firstly, once force could be intensively transferred to mechanophores without being/while being less dispersed to other contrast networks or to align polymer chains, the cleavage of the linkages of mechanophores would be promoted, and the mechanoresponsive sensitivity would be enhanced [19,29,33,44,45]. In the micellar hydrogels, dominant force was intensively transferred to the Rh-crosslinked PMA chains inside microspheres, resulting in a coil-to-stretch transition of polymer chains, and large force was concentrated on Rh mechanophores, favoring the activation of Rh [35]. In comparison, for the elastomer, though the network embedded with Rh mechanophores was a little bit prestretched, limited by its triple crosslinked network structure, when it was stretched, force was largely dispersed to the contrast networks and less was transferred to activating the mechanophores, which resulted in the inferior activation of Rh. Second, an easy-to-reach high orientation state in the direction of stress for polymer chains was also beneficial to the mechanoactivation of mechanophores, resulting in the enhancement of mechanoresponsive sensitivity. In fact, the orientation of polymer chains in hydrogels was easy because water can reduce the glass transition temperature of polymer chains, and further promote the chain-segment movement [53,54]. Moreover, hydrogels here exhibited high fracture strain, with a high strain ratio, and the Rh-crosslinked PMA chains in hydrogels could reach a high alignment state with a low force level, which made contribution to the mechanoactivation of Rh. However, as for the elastomer, since its chain-segment-movement ability was relatively low, it was difficult for the orientation of the polymer chains embedded with Rh in the direction of stress, and, with a low fracture strain, it was also difficult for the polymer chains to achieve a high alignment state, except when a high level of stress was applied. As a result, the mechanoactivation of Rh was weakened accordingly.

Finally, water surrounding the Rh mechanophores in the microspheres was proposed to be beneficial to the enhancement of mechanical sensitivity of Rh as well, and it could promote the activation of Rh induced by force [20]. As a result, compared with that of the elastomer free of water, the mechanoresponsive sensitivity of hydrogels here would be enhanced further. Additionally, the presence of water in microspheres was further probed by the way of pyrene fluorescence (Tables S3 and S4, and Part S2).

**Figure 7:**
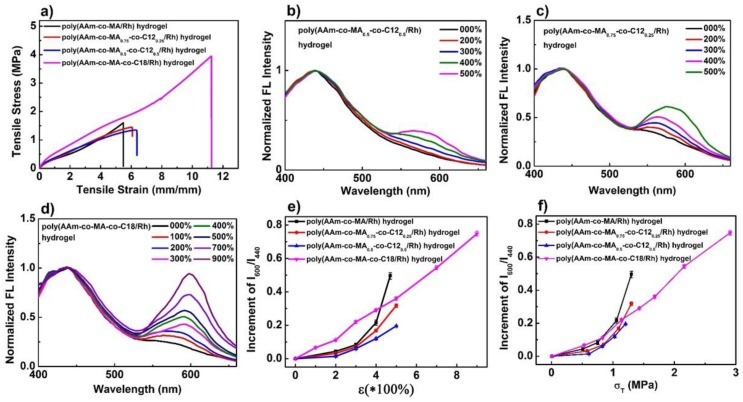
(**a**) The tensile stress–strain curves of poly(AAm-co-MA/Rh) hydrogel (black line), poly(AAm-co-MA_0.75_-co-C12_0.25_/Rh) hydrogel (red line), poly(AAm-co-MA_0.5_-co-C12_0.5_/Rh) hydrogel (blue line), and poly(AM-co-MA-co-C18/Rh) hydrogel (purple line), with a solid content of 70 wt% and the same Rh crosslinker concentration of 0.5 mol% of MA. Emission spectra of (**b**) poly(AAm-co-MA_0.5_-co-C12_0.5_/Rh), (**c**) poly(AAm-co-MA_0.75_-co-C12_0.25_/Rh), and (**d**) poly(AAm-co-MA-co-C18/Rh) hydrogels with a solid content of 70 wt% recorded at increasing strain levels during uniaxial stretching. The increment of fluorescence-intensity ratio *I*_600_/*I*_440_ as a function of (**e**) stretch ratio and (**f**) stress level for the poly(AAm-co-MA/Rh) hydrogel (black line), poly(AAm-co-MA_0.75_-co-C12_0.25_/Rh) hydrogel (red line), poly(AAm-co-MA_0.5_-co-C12_0.5_/Rh) hydrogel (blue line), and poly(AM-co-MA-co-C18/Rh) hydrogel (purple line).

**Figure 8:**
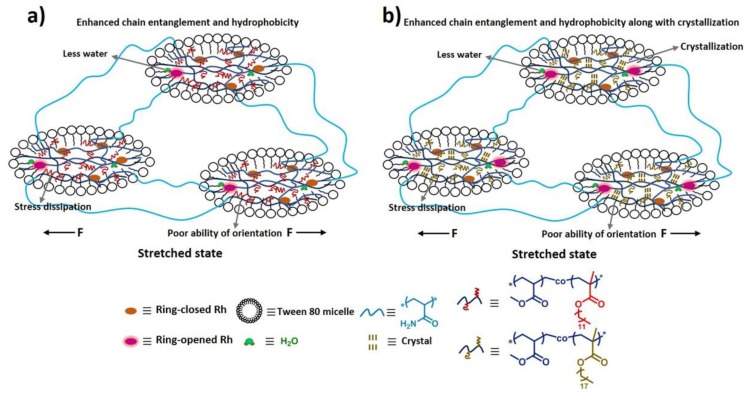
The microstructures of the (**a**) poly(AAm-co-MA-co-C12/Rh) hydrogel and (**b**) partially crystalline poly(AAm-co-MA-co-C18/Rh) hydrogel upon stretching (both of the hydrogels exhibited enhanced chain entanglement and hydrophobicity).

To further confirm the enhancement mechanism of mechanoresponsive sensitivity as mentioned above, control experiments were carried out. As shown in Table S1, two control micellar hydrogels, poly(AAm-co-MA_0.5_-co-C12_0.5_/Rh) (MA_17.5_-C12_17.5_-Rh_0.5_-AAm_35_) and poly(AAm-co-MA_0.75_-co-C12_0.25_/Rh) (MA_26.25_-C12_8.75_-Rh_0.5_-AAm_35_), were fabricated, and their stress–strain curves were obtained, as shown in Figure 7a. We can learn that their mechanical properties were similar to those of the poly(AAm-co-MA/Rh) hydrogel (MA_35_-Rh_0.5_-AAm_35_) (Figure 7a and Table S2), while the gels also presented similar thermomechanical behavior above 0 °C, and none of them showed the behavior of a crystal-phase transition (Figure S8). However, taking poly(AAm-co-MA_0.5_-co-C12_0.5_/Rh) hydrogel as an example, we took its dynamic color change images (Figure S9a and Video S2), and the force-induced color change happened with difficulty, when the stretch ratio varied from 0 to 400%, it did not show an obvious color change unless the stretch ratio exceeded 400%. Accordingly, the emission intensity at 600 nm increased very little with the increase of the stretch ratio (Figure 7b and Figure S10a), and it was really much smaller than that of poly(AAm-co-MA/Rh) hydrogel. Meanwhile, we also measured the dynamic-fluorescence spectra of the poly(AAm-co-MA_0.75_-co-C12_0.25_/Rh) hydrogel, and its emission intensity at 600 nm increased more obviously than that of the poly(AAm-co-MA_0.5_-co-C12_0.5_/Rh) hydrogel (Figure 7c and Figure S10b), but it was still smaller than that of the poly(AAm-co-MA/Rh) hydrogel. Furthermore, we analyzed the relationships of the increment of the fluorescence-intensity ratio (*I*_600_/*I*_440_) and stretch ratio/stress for these three kinds of hydrogels (Figure 7e,f). It can be found that, with the same strain ratio or stress level, the increment of *I*_600_/*I*_440_ for the poly(AAm-co-MA/Rh) hydrogel was higher than the corresponding values for the latter two, and the increment of *I*_600_/*I*_440_ for poly(AAm-co-MA_0.5_-co-C12_0.5_/Rh) hydrogel was the smallest. Thus we can infer that, with the introduction of C12, the mechanical response degree of Rh was diminished; in parallel, the mechanoresponsive sensitivity of the hydrogel was weakened as well, and the more C12 was introduced, the less mechanoresponsive sensitivity would be obtained. In fact, this phenomenon acted as a negative contrast to verify the enhancement mechanism of mechanoresponsive sensitivity. Firstly, the characteristics of the microspheres changed a lot when C12 was introduced, and the main change was that hydrophobic interaction increased dramatically. As a result, the polymer chain entanglement in the microspheres was more serious (Figure 8a). When the hydrogel was stretched, though large force was intensively transferred to the microspheres, the enhanced hydrophobic chain entanglement would disperse large amount, as a result, less force was transferred to the Rh, and the activation of Rh was weakened. Second, to reach a high alignment state for the Rh-crosslinked PMA (C12) chains, and to further induce the cleavage of more linkages of Rh, larger force and more energy should be borne and dissipated through the deformation of microspheres, then higher tensile strain and stronger mechanical strength were needed; in fact, limited by the mechanical properties, it was difficult for the polymer chains to reach a high alignment state, and the linkages of Rh fractured difficultly, so the activation of Rh was diminished. In a word, due to the low efficiency of stress concentration on Rh and the difficulty of aligning the Rh-crosslinked polymer chains in a high orientation state, the mechanoresponsive sensitivity of the hydrogels loaded with an amount of C12 were obviously weakened. Moreover, with the introduction of C12 into microspheres, the ratios *I*_1_/*I*_3_ for the poly(AAm-co-MA-co-C12/Rh) hydrogel were smaller than that in the poly(AAm-co-MA/Rh) hydrogel (Table S4), which meant microspheres fabricated by MA and C12 were more hydrophobic, and Rh in the microspheres was surrounded by much less water. Therefore, the positive effects of water were diminished, and, as a result, the sensitivity to mechanical stress was weakened accordingly.

Interestingly, we also fabricated another micellar hydrogel, poly(AAm-co-MA-co-C18/Rh) (MA_17.5_-C18_17.5_-Rh_0.5_-AAm_35_) (Table S1), and it was extremely strong and robust (Figure 7a, Table S2). The most important factor to improve its mechanical properties was that there existed alkyl crystals that were created by the hexagonal packing of side alkyl chains of C18 segments [55,56]. A DSC measurement was carried out, and the melting and crystallization peaks clearly emerged in the DSC scans (Figure S11). In parallel, excellent dynamic color-change images were taken (Figure S9b and Video S3); when the stretch ratio reached only 200%, an obvious color change emerged, and with the increase of strain ratio, the red coloration gradually deepened. We measured its dynamic fluorescence spectra (Figure 7d), and the relationships of stress, fluorescence intensity, and stretch ratio were plotted in Figure S10c. The emission intensity at 600 nm increased obviously with the increase of strain, and the maximum intensity ratio this hydrogel could reach was as high as 0.94, which was much higher than that of the poly(AAm-co-MA/Rh) hydrogel. Thanks to the crystallization-enhanced mechanical property, the mechanical response degree of Rh was greatly increased, and the highest mechanical response degree of Rh this hydrogel could reach was much higher.

Importantly, this hydrogel could also be used as a control sample to verify the mechanism of the enhancement of mechanoresponsive sensitivity in some degree. As shown in Figure 7f, when the stress applied was less than 0.8 MPa, with the same strain ratio or stress level, the increment of *I*_600_/*I*_440_ for this hydrogel was higher than that for the poly(AAm-co-MA/Rh) hydrogel, which was associated with the fact that crystallization could slow the stress relaxation of the polymer chains, thus promoting the orientation of the Rh crosslinked soft segments, and further enhancing mechanoresponsive sensitivity [30]. However, within the range of higher stress levels, the increment of *I*_600_/*I*_440_ for this hydrogel turned obvious lower than that for the poly(AAm-co-MA/Rh) hydrogel, which was because with a higher strain ratio/stress level, the effect of crystallization was weakened, and the effect of water and energy dissipation turned more important, which resulted in the decrease of mechanoresponsive sensitivity (Figure 8b). So, it can be learned that, although the presence of crystallization had a negative impact on the confirmation of our enhancement mechanism with a higher stress ratio/stress level, this hydrogel could also be used to verify our mechanism, and the corresponding discussion was quite similar to that for the poly(AAm-co-MA-co-C12/Rh) hydrogel (Figure 8).

### 3.6. Acid/Alkali-Responsive Properties of Rh-Functionalized Hydrogels

**Figure 9:**
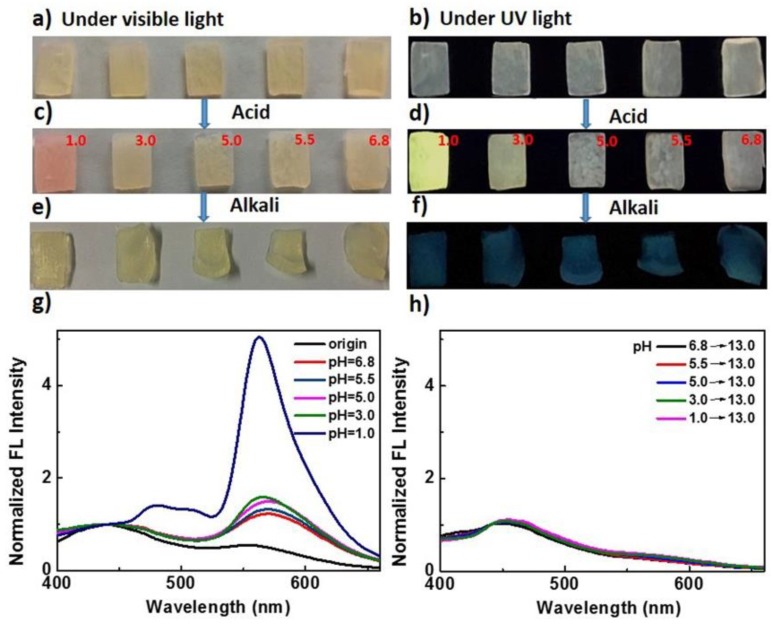
The optical (**a**) and fluorescent images (**b**) of the original poly(AAm-co-MA/Rh) hydrogels with a solid content of 70 wt%. The (**c**) optical and (**d**) fluorescent images of the hydrogels after swelling in water solutions with different pH (The pH values were noted in the images). The corresponding (**e**) optical and fluorescent (**f**) images of the hydrogels after further swelling in the alkaline solutions with a pH of 13.0. Emission spectra of the hydrogels (**g**) after swelling in water solutions with different pH values from 1.0 to 6.8 and (**h**) after further swelling in the alkaline solutions with a pH of 13.0.

We also studied the pH responsiveness of the hydrogels based on the Rh mechanophore. Taking 70 wt% poly(AAm-co-MA/Rh) hydrogel as an example, the acidichromic behaviors of Rh incorporated in the microspheres were investigated. Five pieces of hydrogels were immersed into water solutions with different pH values, from 1.0 to 6.8. The original optical and fluorescent images are shown in Figure 9a,b. After swelling for 36 h, their swelling ratios were almost the same (Table S5), while the changes of the optical and fluorescent color emerged, especially for that in a water solution with a pH of 1.0, of which the change was extremely obvious (Figure 9c,d). Meanwhile, we examined the fluorescence spectra of the hydrogels after swelling in the solutions with different pH values. As shown in Figure 9g, compared with the fluorescence spectra of original hydrogel, the emission intensity of the broad band ranging from 520 to 625 nm increased for all of the swollen hydrogels; however, when the pH varied from 6.8 to 3.0, the corresponding increments of fluorescence intensity were all small. In contrast, when the pH was 1.0, ultrahigh emission intensity centered at 550 nm emerged in the corresponding fluorescence spectrum. It can be learned that the amounts of Rh activated by acid when pH was 1.0 was much more than others’, and the pH needed to trigger the obvious acidichromic property of Rh in hydrogel was much lower than reported before [50,51,52]. This was possibly because the diffusion of H^+^ into the microspheres was difficult; only when the pH was 1.0 was the concentration gradient of H^+^ outside and inside the microspheres high enough, and certain amounts of H^+^ diffused into the microspheres. As a result, much Rh was activated. Furthermore, all the hydrogels were further immersed into water solutions with a pH of 13.0. After swelling for 5 h, the optical color of all hydrogels got blonder, and the fluorescent color all changed to be blue (Figure 9e,f). Accordingly, the fluorescence intensity of the broad band ranging from 520 to 625 nm decreased dramatically (Figure 9h). It can be concluded that the colored and fluorescent Rh amide form in the micellar hydrogels could also reverse back to the colorless and nonfluorescent spirolactam form with the addition of alkali.

## 4. Conclusions

In conclusion, a novel mechanochromic and mechanofluorescent hydrogel based on a Rh mechanophore was developed by micellar-copolymerization of Rh crosslinker in the presence of methyl acrylate and acrylamide within a uniform emulsion. Within the micellar hydrogel, mechanoresponsive sensitivity was enhanced greatly, because large force could be concentrated within the microspheres and further effectively transferred to Rh, and the Rh-crosslinked polymer chains in the microspheres could be stretched to a high alignment state easily. Both promoted the cleavage of the linkages of Rh mechanophores, while the water surrounding the Rh mechanophores made a large contribution to the activation of the Rh spirolactam system. As a result, the stress needed to trigger the mechanochromic properties of Rh in hydrogel was much lower than in its native polymer matrix reported before. The hydrogel based on Rh, therefore, exhibited excellent mechanochromic properties even at lower stress. Meanwhile, the visible and fluorescent color changes were reversible; thus, the hydrogels could be used as reusable materials for color printing/writing. Of peculiar importance was that the hydrogel could emit highly bright fluorescence under sufficient stress or strain, and, since the fluorescence signal output is ratiometric and almost free from external interference, the resulting Rh-based hydrogels with high quantum efficiency could be used to detect stress/strain quantificationally through the calculation of fluorescence data, which were more accurate and effective than those through the analysis of qualitative color changes. Surprisingly, the mechanical-response degree of Rh incorporated in the micellar hydrogels containing C18 segments was increased greatly, which was ascribed to the excellent mechanical properties resulting from the formation of lamellar crystals. Finally, we also found that the hydrogel could respond to acid/alkali and exhibited outstanding properties of acidichromism and acidifluorochromism, but the pH needed to trigger the obvious acidichromic property of Rh in hydrogel was much lower than reported before, mainly because the micelles into which Rh was embedded prevented the diffusion of hydrogen ions in hydrogel considerably. The Rh-functionalized hydrogels fabricated here expanded the mechanochromic hydrogel system, and, as double stimuli-responsive hydrogels, they have potential applications in the fields of damage, stress/strain, or acid/alkali detection special by fluorescence.

## Supplementary Materials

The following are available online at http://www.mdpi.com/2073-4360/10/9/994/s1, Scheme S1: Synthetic procedures of the Rh crosslinker, Figure S1: ^1^H NMR spectrum of Rh crosslinker, Table S1: Composition of poly(AAm-co-MA/Rh) gels prepared, Table S2: Mechanical properties of poly(AAm-co-MA/Rh) gels prepared, Figure S2: Strain-induced dynamic color change of 50 wt% and 60 wt% hydrogels, Figure S3: Successive cyclic loading curves of poly(AAm-co-MA/Rh) hydrogels, Figure S4: Stress and fluorescence-intensity ratio as a function of stretch ratio for the 50 wt% and 60 wt% hydrogels, Figure S5: The experimental setup for pen-writing test, Figure S6: Dynamic fluorescence decay of poly(AAm-co-MA/Rh) hydrogel after removing stress, Figure S7: Relative intensity ratio *I*_600_/*I*_440_ as a function of the repeated cycle of stretching and relaxing, Part S1: Reversibility of mechanofluorochromism of the Rh-based hydrogels, Table S3: Composition of poly(AAm-co-MA/EGDMA+Py) gels, Table S4: Relative band intensities *I*_1_/*I*_3_ for pyrene fluorescence, Part S2: Probing the polarity of the microspheres in hydrogels, Figure S8: DSC scans of poly(AAm-co-MA-co-C12/Rh) and poly(AAm-co-MA-co-C18/Rh) hydrogels, Figure S9: Strain-induced dynamic color change of poly(AAm-co-MA-co-C12/Rh) and poly(AAm-co-MA-co-C18/Rh), Figure S10: Stress and fluorescence intensity ratio *I*_600_/*I*_440_ as a function of stretch ratio for the poly(AAm-co-MA-co-C12/Rh) hydrogel and poly(AAm-co-MA-co-C18/Rh) hydrogel, Figure S11: DSC scans of poly(AAm-co-MA-co-C18/Rh) hydrogels, Table S5: Equilibrium-swelling ratios of hydrogels immersed into the water solutions with different pH values, Video S1: Dynamic color change of poly(AAm-co-MA-co/Rh) hydrogel, Video S2: Dynamic color change of poly(AAm-co-MA_0.5_-co-C12_0.5_/Rh), Video S3: Dynamic color change of poly(AAm-co-MA-co-C18/Rh) hydrogel.
