# Supplementary material for: Rhodamine-Functionalized Mechanochromic and Mechanofluorescent Hydrogels with Enhanced Mechanoresponsive Sensitivity
Source: Polymers (Basel). 2018 Sep 6;10(9):994. doi: 10.3390/polym10090994 (PMC6403975; doi:10.3390/polym10090994)
Supplement: Supplementary file 1 [file polymers-10-00994-s001.pdf]

Supporting Information

Rhodamine-Functionalized Mechanochromic and Mechanofluorescent Hydrogels with Enhanced Mechanoresponsive Sensitivity

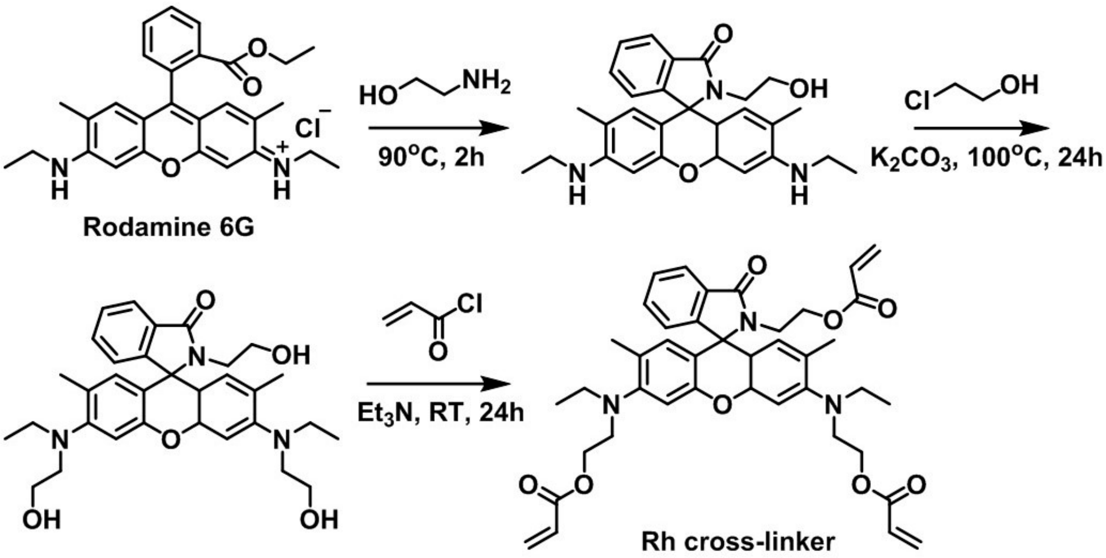

Scheme S1. Synthetic procedures of the Rh crosslinker.

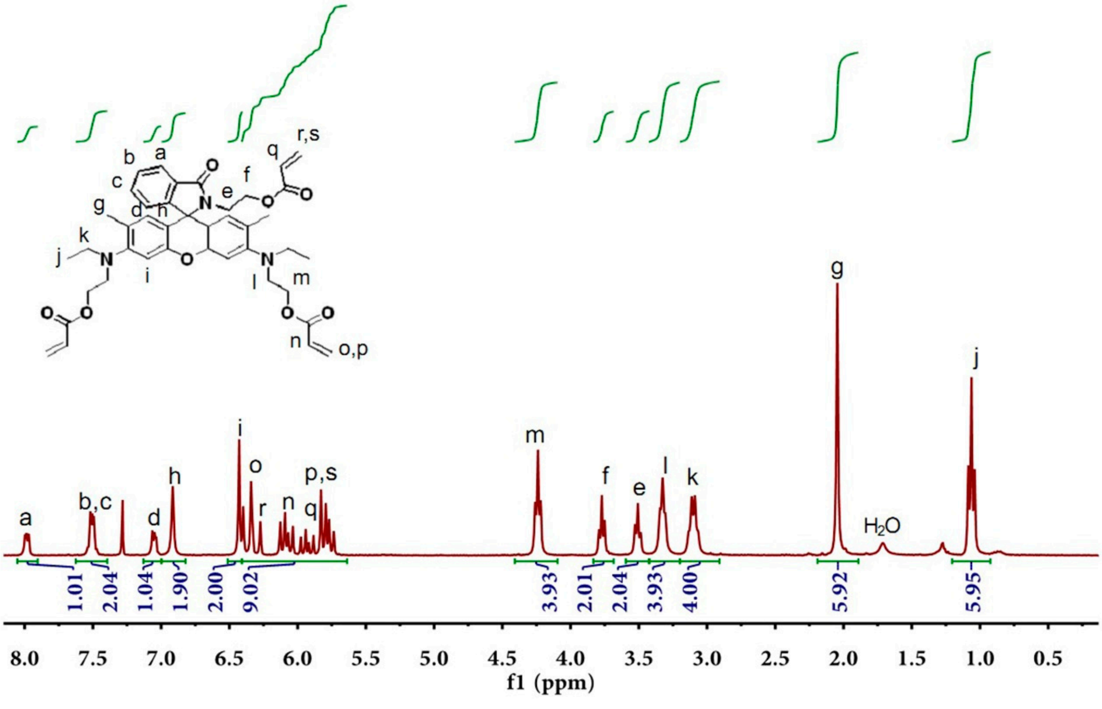

Figure S1. ^1H NMR spectrum of Rh crosslinker (solvent $CDCl_3$, 400MHz).

Table S1. A summary of the composition of poly(AAm-co-MA/Rh) gels prepared at different conditions.

Gel Sample[a]	Water content (wt%)	MA content (wt%)	C12 content (wt%)	C18 content (wt%)	Rh Content (mg)
**MA_{25}-$Rh_{0.3}$-AAm_{25}**	50	25	-	-	12.5
**MA_{25}-$Rh_{0.4}$-AAm_{25}**	50	25	-	-	16.7
**MA_{25}-$Rh_{0.5}$-AAm_{25}**	50	25	-	-	20.8
**MA_{25}-$Rh_{0.6}$-AAm_{25}**	50	25	-	-	25.0
**MA_{30}-$Rh_{0.3}$-AAm_{30}**	40	30	-	-	14.4
**MA_{30}-$Rh_{0.4}$-AAm_{30}**	40	30	-	-	19.2
**MA_{30}-$Rh_{0.5}$-AAm_{30}**	40	30	-	-	24.0
**MA_{30}-$Rh_{0.6}$-AAm_{30}**	40	30	-	-	28.8
**MA_{35}-$Rh_{0.3}$-AAm_{35}**	30	35	-	-	16.8
**MA_{35}-$Rh_{0.4}$-AAm_{35}**	30	35	-	-	22.4
**MA_{35}-$Rh_{0.5}$-AAm_{35}**	30	35	-	-	28.0
**MA_{35}-$Rh_{0.6}$-AAm_{35}**	30	35	-	-	33.6
**$MA_{17.5}$-$C12_{17.5}$-$Rh_{0.5}$-AAm_{35}**	30	17.5	17.5	-	28.0
**$MA_{26.25}$-$C12_{8.75}$-$Rh_{0.5}$-AAm_{35}**	30	26.25	8.75	-	28.0
**$MA_{17.5}$-$C18_{17.5}$-$Rh_{0.5}$-AAm_{35}**	30	17.5	-	17.5	28.0

a) MAx-C12(C18)y-Rhz-AAmt: x is a MA concentration (wt%); y is a C12 or C18 concentration (wt%); z is a molar ratio of Rh to MA (mol%); t is a AAm concentration (wt%).

Table S2. A summary of the mechanical properties of poly(AAm-co-MA/Rh) gels prepared at different conditions.

Gel Sample[a]	Water content (wt%)	E (kPa)	σ_f (MPa)	ε_f (mm/mm)
**MA$_{25}$-Rh$_{0.3}$-AAm$_{25}$**	50	176	0.59	11.6
**MA$_{25}$-Rh$_{0.4}$-AAm$_{25}$**	50	188	0.72	11.0
**MA$_{25}$-Rh$_{0.5}$-AAm$_{25}$**	50	419	0.92	10.5
**MA$_{25}$-Rh$_{0.6}$-AAm$_{25}$**	50	431	0.78	8.2
**MA$_{30}$-Rh$_{0.3}$-AAm$_{30}$**	40	200	0.82	8.9
**MA$_{30}$-Rh$_{0.4}$-AAm$_{30}$**	40	234	0.90	8.0
**MA$_{30}$-Rh$_{0.5}$-AAm$_{30}$**	40	431	1.20	6.8
**MA$_{30}$-Rh$_{0.6}$-AAm$_{30}$**	40	462	1.04	5.7
**MA$_{35}$-Rh$_{0.3}$-AAm$_{35}$**	30	718	1.33	8.4
**MA$_{35}$-Rh$_{0.4}$-AAm$_{35}$**	30	374	1.47	6.8
**MA$_{35}$-Rh$_{0.5}$-AAm$_{35}$**	30	632	1.60	5.6
**MA$_{35}$-Rh$_{0.6}$-AAm$_{35}$**	30	1075	1.37	4.5
**MA$_{17.5}$-C12$_{17.5}$-Rh$_{0.5}$-AAm$_{35}$**	30	762	1.35	6.4
**MA$_{26.25}$-C12$_{8.75}$-Rh$_{0.5}$-AAm$_{35}$**	30	701	1.45	6.0
**MA$_{17.5}$-C18$_{17.5}$-Rh$_{0.5}$-AAm$_{35}$**	30	1166	3.95	11.2

a) MAx-C12(C18)y-Rhz-AAmt: x is a MA concentration (wt%); y is a C12 or C18 concentration (wt%); z is a molar ratio of Rh to MA (mol%); t is a AAm concentration (wt%).

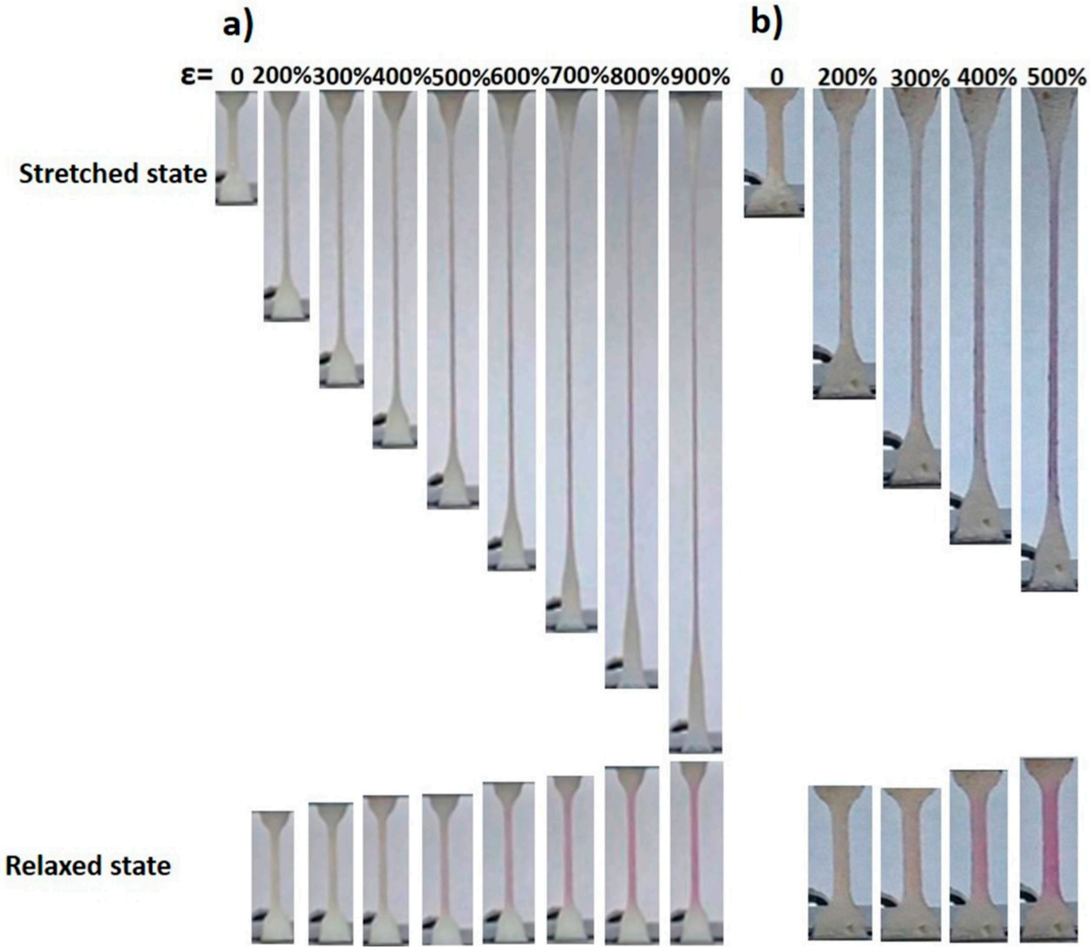

Figure S2. Strain-induced dynamic color change of poly(AAm-co-MA/Rh) hydrogels with solid contents of **a)** 50 wt% and **b)** 60 wt% (with a fixed Rh crpsslinker concentration of 0.5 mol% of MA) at both stretched state (stretch ratios of 000%–900% or 500%) and the corresponding relaxed state.

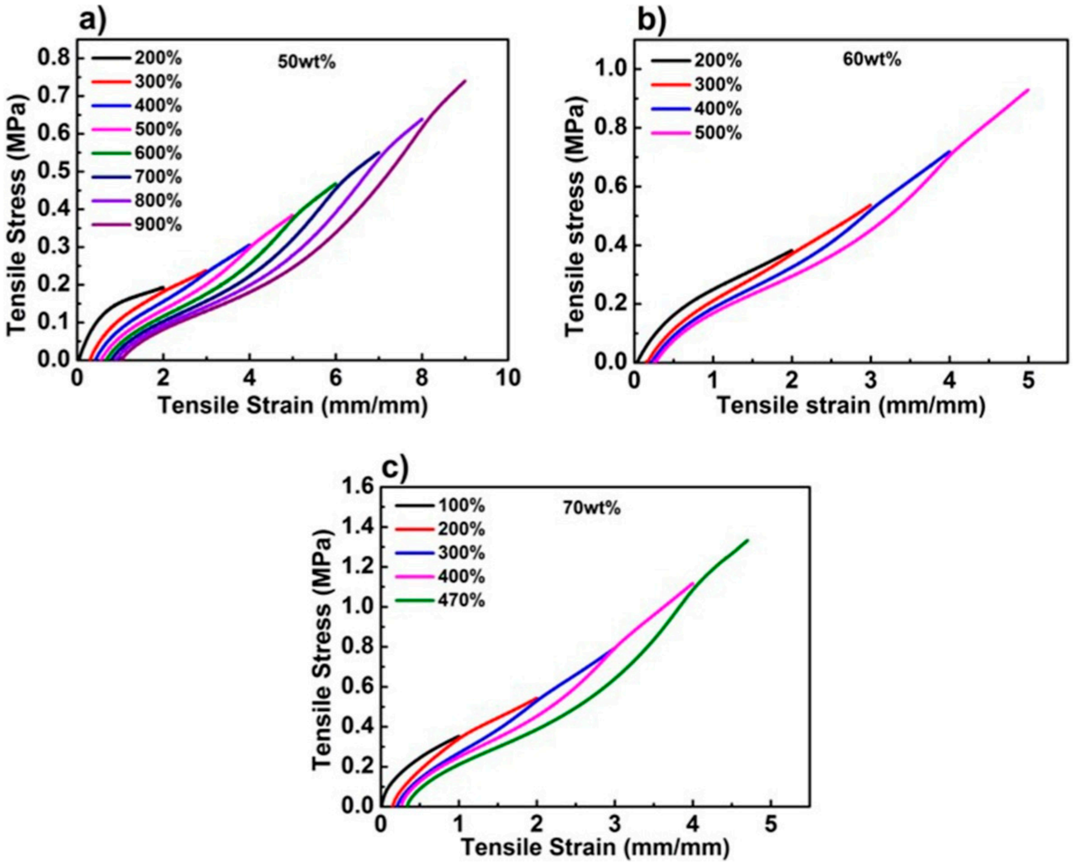

Figure S3. Successive cyclic loading curves of poly(AAm-co-MA/Rh) hydrogels with different solid contents **a)** 50 wt%, **b)** 60 wt%, **c)** 70 wt% at a fixed Rh crsslinker concentration of 0.5 mol% of MA. The stretching rate was 60 mm/min for loading and unloading cycles.

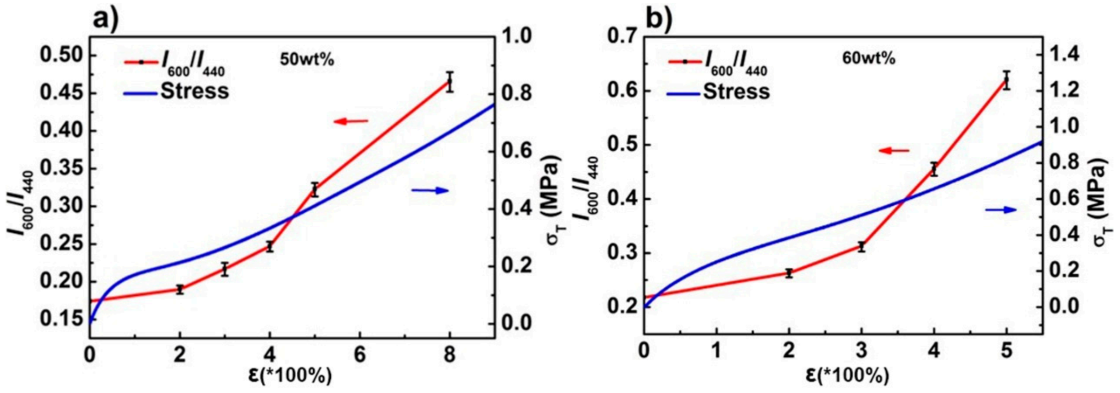

Figure S4. Stress and fluorescence intensity ratio I_{600}/ I_{440} as a function of stretch ratio for the hydrogel with a solid content of **a)** 50 wt% and **b)** 60 wt%.

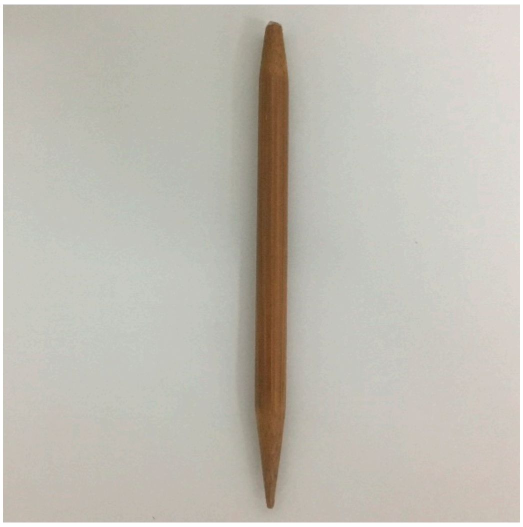

Figure S5. The experimental setup for pen-writing test.

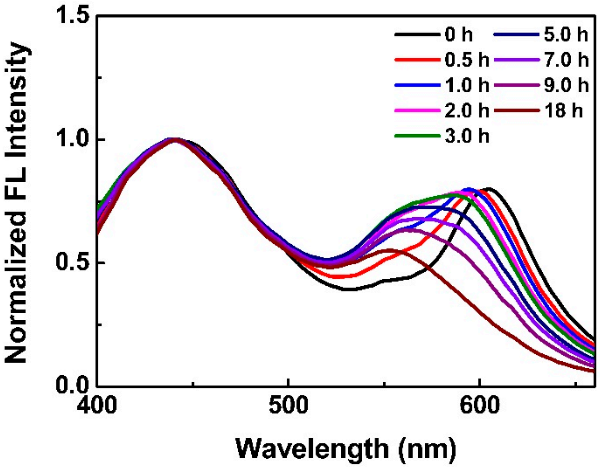

Figure S6. Fluorescence spectra of 70 wt% poly(AAm-co-MA/Rh) hydrogel with different times after removing stress.

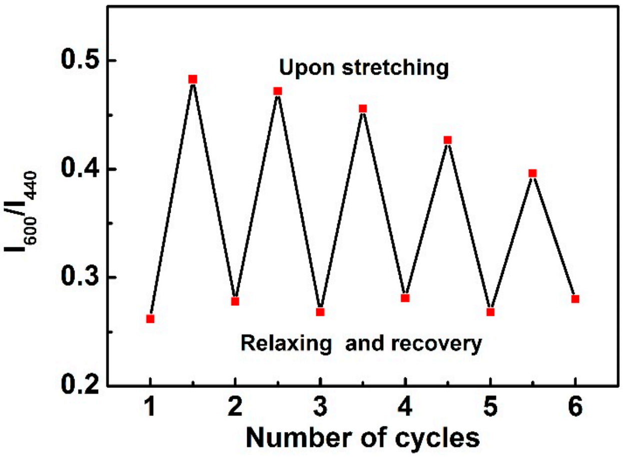

Figure S7. Relative intensities of the emission at 600 and 440 nm of 70 wt% hydrogel as a function of the repeated cycle of stretching (the stretch ratio is 400%) and relaxing (the gels undergo a color-recovery process).

Part S1. The Reversibility of Mechanofluorochromism of the Rh Based Hydrogels.

According to the previous work,[1] the ring-opening Rh mechanophores in the triple-networks elastomer could almost all go automatically back to its ring-closed form within 5 h at room temperature. Nevertheless, it was beyond our expectation that the dynamic recovery process was sustained for a long period of time, over 18 h (**Figure S5**). As shown in **Figure S5**, for the 70 wt% hydrogel with a prestretching strain of 470%, after the stress was released, the emission peak wasn't blue-shifted dramatically and it was still centered near 600 nm, which highly agreed with the phenomenon that the relaxed gels hold a noticeably pink color instead of yellow. This finding was quite different from the previous results; however, it was extremely reasonable and explicable. This phenomenon was probably associated with the hydrogel structure when the stress was released, the stretched Rh-crosslinked chains couldn't go back to their original tangled state immediately, and the deformation of microspheres could not entirely be removed in a short time; therefore, there still existed small residual network deformation and, as a result, residual stress was still distributed along the PMA chains. With the extension of time, firstly, the fluorescence emission peak was blue-shifted gradually, and a new broad emission peak around 560 nm emerged and the corresponding intensity increased gradually, while in parallel, the emission intensity at 600 nm decreased continually. After about 5 h, the boundary between the two emission peaks was not clear, and with the time further prolonged, the emission intensity around 560 nm and that at 600 nm both decreased slowly. After 18h, a single emission peak at 550 nm emerged, however, both the visible color and the fluorescent color of the gel didn't fade away absolutely but still emerged to be a little red. These were all attributed to the special micellar structure incorporated into the hydrogel, which caused the delay of stress relaxation of the polymer chains. As a result, much longer time was needed to reach a new dynamical equilibrium.[2]

In addition, the force-induced emission "on–off" switch could be repeated for multiple cycles with a certain degree of change in the intensity ratio I_{600}/I_{440}. The emission "on" presented the mechanochromic/mechanofluorescent process, and the emission "off" reflected the process that the gel went back to its colorless state. Although the relative intensity ratio I_{600}/I_{440} upon stretching repeatedly decreased gradually, it still could be used to verify the fact that the corresponding visible and fluorescent color changes were reversible.

Table S3. A summary of the composition of poly(AAm-co-MA/EGDMA+Py) gels prepared at different conditions.

Gel Sample[a]	Water content (wt%)	MA content (wt%)	C12(C18) content (wt%)	EGDMA Content (mg)
**MA_{25}-$EM_{0.75}$-AAm_{25}**	50	25	-	8.7
**MA_{30}-$EM_{0.75}$-AAm_{30}**	40	30	-	10.1
**MA_{35}-$EM_{0.75}$-AAm_{35}**	30	35	-	11.7
**$MA_{17.5}$-$C12_{17.5}$-$EM_{0.75}$-AAm_{35}**	30	17.5	17.5	11.7
**$MA_{26.25}$-$C12_{8.75}$-$EM_{0.75}$-AAm_{35}**	30	26.25	8.75	11.7
**$MA_{17.5}$-$C18_{17.5}$-$EM_{0.75}$-AAm_{35}**	30	17.5	17.5	11.7

a) MAx-C12(C18)y-EMz-AAmt: x is a MA concentration (wt%); y is a C12 or C18 concentration (wt%); z is a molar ratio of EGDMA to MA (mol%); t is a AAm concentration (wt%).

Table S4. Summary of relative band intensities I_1/I_3 for pyrene fluorescence in various hydrogels and PMA.

Samples	MA_{25}-$EM_{0.75}$-AAm_{25}	MA_{30}-$EM_{0.75}$-AAm_{30}	MA_{35}-$EM_{0.75}$-AAm_{35}	$MA_{17.5}$-$C12_{17.5}$-$EM_{0.75}$-AAm_{35}	$MA_{17.5}$-$C18_{17.5}$-$EM_{0.75}$-AAm_{35}	$MA_{17.5}$-$C18_{17.5}$-$EM_{0.75}$-AAm_{35}	PMA
I_1/I_3	1.053	0.888	0.886	0.826	0.837	0.853	0.668

Part S2. Probing the Polarity of the Microspheres in Hydrogels.

Water diffusing into the microspheres was probed by the way of pyrene fluorescence, and the pyrene was introduced into several hydrogels by first dissolving it in the MA (Table S3). Since the pyrene was highly hydrophobic, almost all pyrene molecules were excited in the microspheres in the hydrogels. As is well known, the intensity ratio (I1/I3) of the first over the third vibronic band of the emission spectrum of pyrene is sensitive to the polarity of the microenvironment3; here, the values of the ratio I1/I3 for hydrogels and pure crosslinked PMA were calculated (Table S4). The ratio I1/I3 for the pyrene fluorescence in water was about 1.85,4, 5 and as shown in Table S4, the ratios I1/I3 for the pyrene fluorescence in hydrogels varied from 0.83 to 1.05, which were all obviously smaller than that in water. In fact, it precisely reflected the hydrophobicity of the microspheres where pyrene was located in. Moreover, the ratio I1/I3 for the pyrene fluorescence in pure crosslinked PMA was 0.67, and it was smaller than all of that in hydrogels; therefore, we can learn that the polarity of the microenvironment that pyrene was located in was higher than that in pure PMA, which meant that pyrene in the microspheres was actually surrounded by a small amount of water, and water diffusing into the microspheres within hydrogels could be verified.

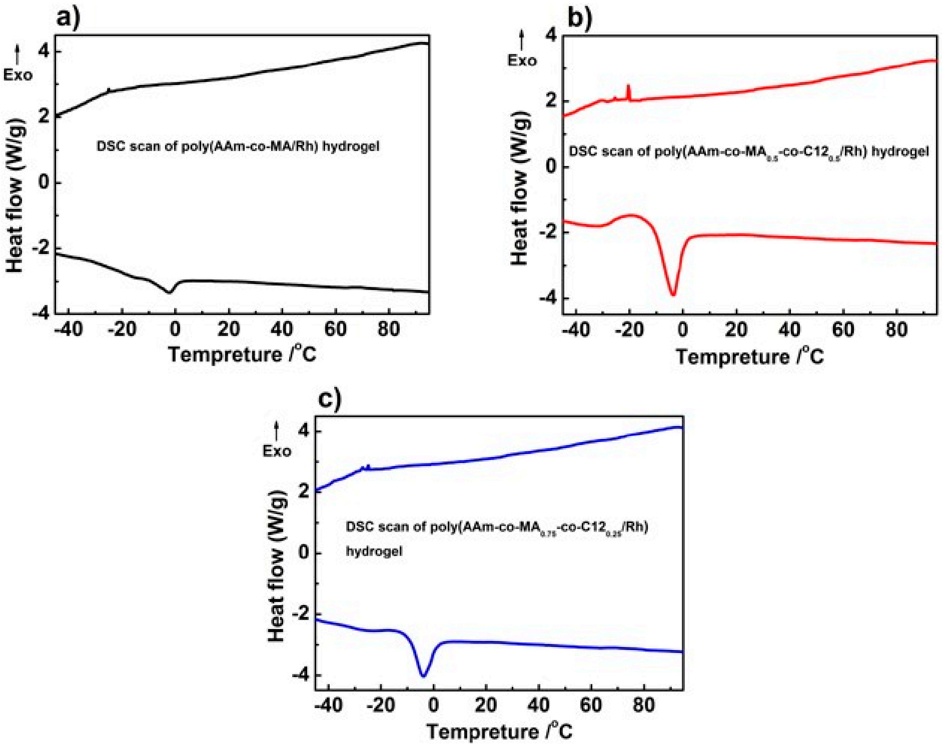

Figure S8. DSC scans of **a)** poly(AAm-co-MA/Rh) hydrogel, **b)** poly(AAm-co-$MA_{0.5}$-co-$C12_{0.5}$/Rh) hydrogel and **c)** poly(AAm-co-$MA_{0.75}$-co-$C12_{0.25}$/Rh) hydrogel (None of them showed crystal-phase transition).

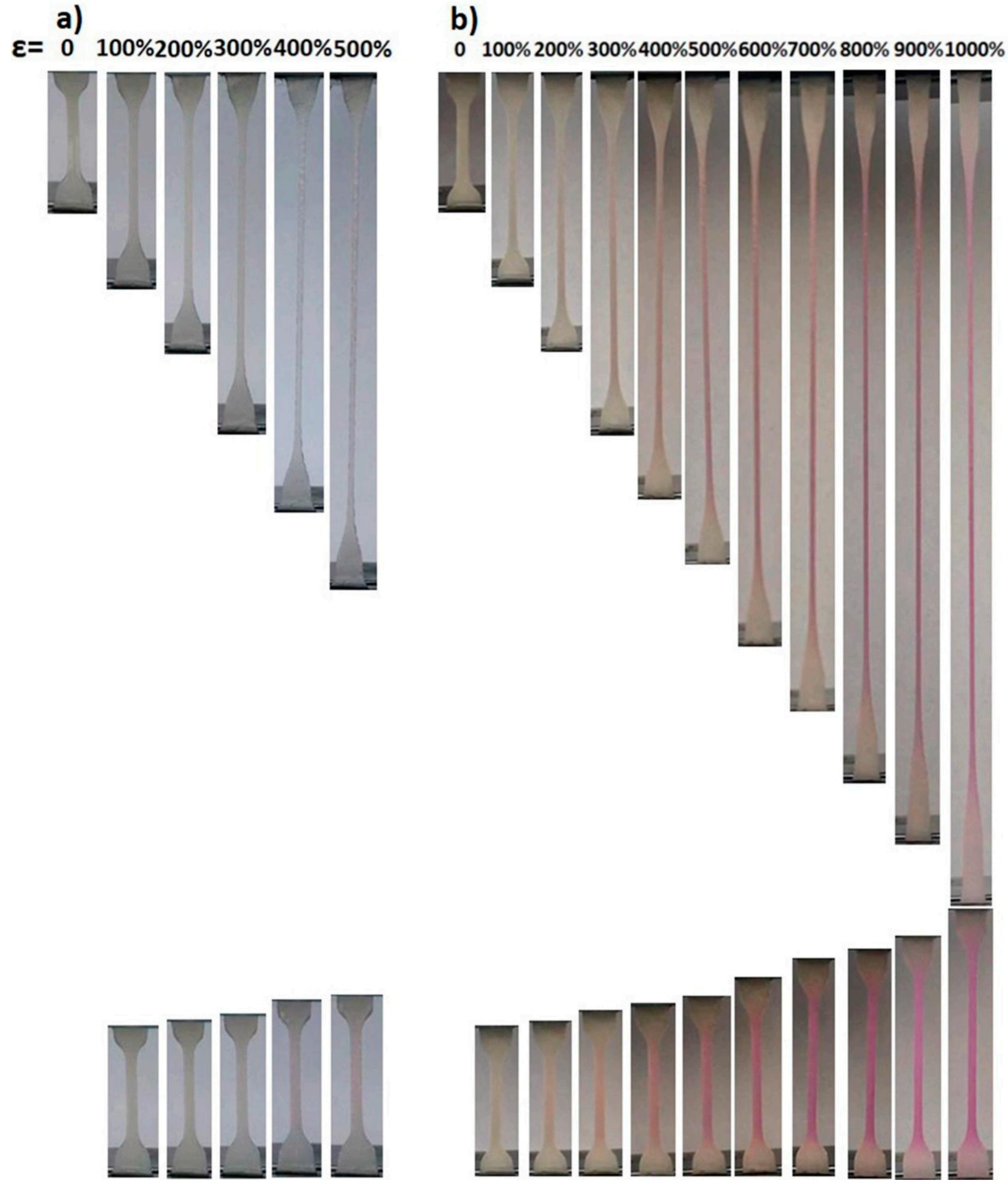

Figure S9. Optical images of **a)** poly(AAm-co-$MA_{0.5}$-co-$C12_{0.5}$/Rh) and **b)** poly(AAm-co-MA-co-C18/Rh) with a solid content of 70 wt% at both stretched state and the corresponding relaxed state.

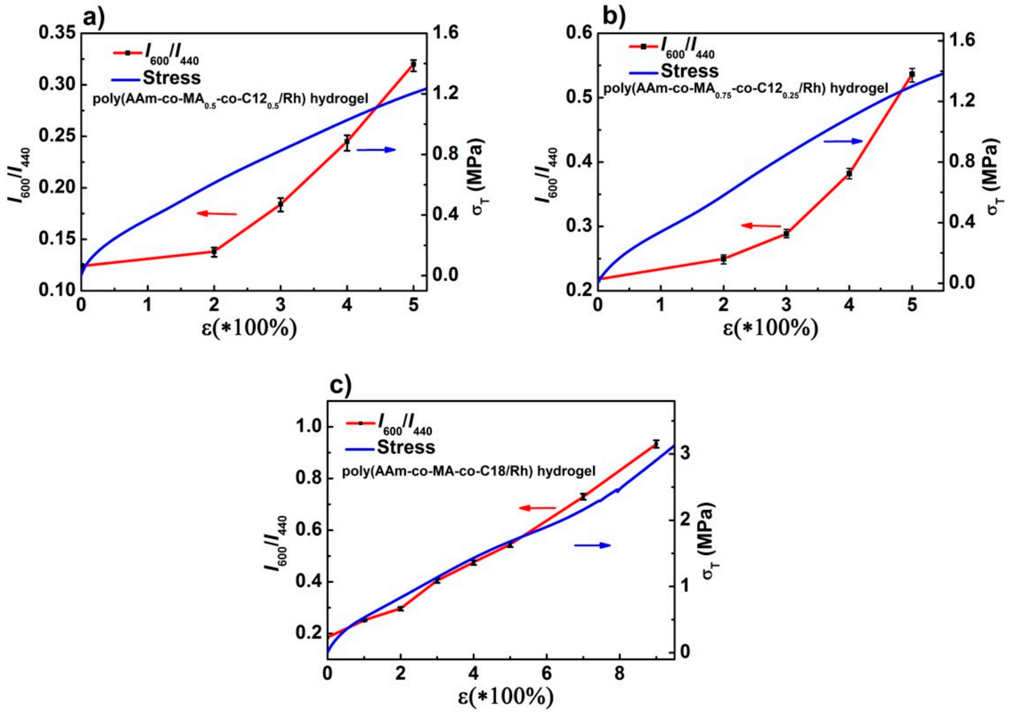

Figure S10. Stress and fluorescence-intensity ratio I_{600}/I_{440} as a function of stretch ratio for a) the poly(AAm-co-MA$_{0.5}$-co-C12$_{0.5}$/Rh) hydrogel, b) poly(AAm-co-MA$_{0.75}$-co-C12$_{0.25}$/Rh) hydrogel and c) poly(AAm-co-MA-co-C18/Rh) hydrogel with a solid content of 70 wt%.

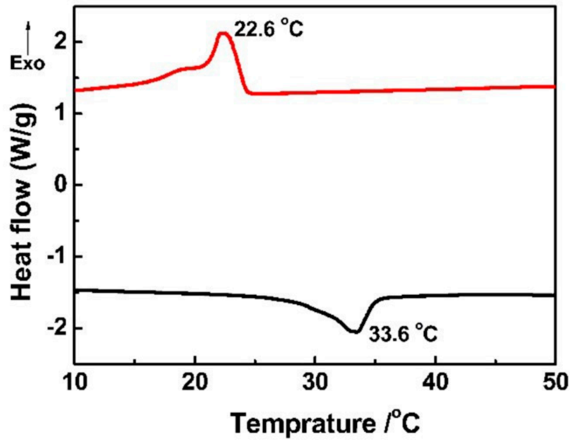

Figure S11. DSC scans of poly(AAm-co-MA-co-C18/Rh) hydrogels (the melting and crystallization temperatures are also indicated).

Table S5. Simplified equilibrium swelling ratios (ESRs) of hydrogels immersed into the water solutions with different pH values.

**pH**	1.0	3.0	5.0	5.5	6.8
**Swelling ratio**	1.96	1.90	1.95	1.96	2.01

Note: ESR=Ws/Wo, Ws means the weight of the hydrogel after swelling equilibrium, and Wo means the weight of the original hydrogel before swelling.

References

1. T.-S. Wang, N. Zhang, J.-W. Dai, Z.-L. Li, W. Bai and R.-K. Bai, *ACS Appl. Mater. Interfaces*, **2017**, *9*, 11874–11881.
2. L.-J. Wang, X.-J. Zhou, X.-H. Zhang and B.-Y. Du, *Macromolecules*, **2015**, *49*, 98–104.
3. S. Farhangi and J. Duhamel, *J. Phys. Chem. B*, **2016**, *120*, 834–842.
4. K. Kalyanasundaram and J. K. Thomas, *J. Am. Chem. Soc.*, **1977**, *99*, 2039–2044.
5. D. C. Dong and M. A. Winnik, *Can. J. Chem.*, **1984**, *62*, 2560–2565.
